# Life of a droplet: Buoyant vortex dynamics drives the fate of micro-particle expiratory ejecta

**DOI:** 10.1063/5.0032591

**Published:** 2020-12-01

**Authors:** E. Renzi, A. Clarke

**Affiliations:** Department of Mathematical Sciences, Loughborough University, Loughborough LE11 3TU, United Kingdom

## Abstract

We show that the dynamics of the expiratory cloud ejected during human respiratory events can be modeled by extending the theory of buoyant vortex rings with an initial momentum. We embed the integral conservation laws that govern the cloud’s motion into the model of an expanding vortex to determine the velocity field inside and outside the cloud. We then apply a Lagrangian particle-tracking model to calculate the trajectories of the mucosalivary droplets suspended within the cloud. Our results show very good agreement with the available experimental data. The vortex is shown to have a significant effect on suspending the droplets present in the cloud, increasing the time they remain airborne and extending their range further than predicted by the existing models. We also study the role that initial conditions have on the maximum streamwise range of the droplets, finding that decreasing the angle of projection can reduce the spread of the droplets by an order of meters. Finally, we discuss the importance of these findings in the context of informing public health policies and global information campaigns to slow down the spread of respiratory viruses.

## I. INTRODUCTION

In this paper, we develop a physico-mathematical characterization of the vortex dynamics of expiratory clouds based on experimental evidence and quantify how this dynamics affects the fate of the droplets ejected during human respiratory events.

The ongoing Coronavirus Disease 2019 (COVID-19) pandemic caused by the Severe Acute Respiratory Syndrome Coronavirus 2 (SARS-CoV-2) has shown how respiratory viruses are able to spread across an entire continent, leaving us unprepared. That is because we have only a basic knowledge of their spreading mechanisms. While the epidemiologic and pathogenetic aspects of the problem are now determined to a good degree of confidence,^1–3^ the fluid dynamics governing the dispersal of virus-laden mucosalivary droplets is still puzzling.

The mucosalivary droplets are made of oral matter that is ejected via the respiratory system during expiratory events, such as breathing, coughing, and sneezing. If these droplets contain viable virus particles (virions), they can transmit the virus from an infected individual to a susceptible host through (i) direct deposition on the mucous membranes, (ii) inhalation of long-range airborne virus particles, and (iii) contamination of objects.^1,4^ Until recently, respiratory droplets were classified into two distinct categories: “large” droplets, which follow a ballistic trajectory and contaminate surfaces and people nearby, and “small” droplets, which evaporate into droplet nuclei (aerosols).^5^ The cut-off diameter used in the literature to discriminate between large and small droplets ranges from 5 *μ*m up to 150 *μ*m, depending on the ambient temperature and relative humidity (RH).^1,2^ The social distancing policies adopted in many parts of the world to mitigate the ongoing COVID-19 crisis are precisely based on this concept.

However, the seminal investigation of the Bourouiba Lab at MIT has recently highlighted the important misconceptions in the existing droplet classification. References 1 and 6 provide experimental evidence that human respiratory ejecta are not made of isolated droplets, rather they are a continuous, turbulent multiphase cloud of buoyant gas, carrying suspended droplets of differing sizes. The moist atmosphere of the cloud dramatically slows down evaporation and increases the lifetime of the droplets by a factor 1000, from fractions of a second to minutes.^1,6^ The dynamics of this turbulent flow is much more complex than the dichotomic droplet model and is still poorly understood.

Only very recently have scientists started to investigate the properties of the expiratory cloud, mostly focusing on predicting the maximum distance traveled by the droplets.^6–12^ Investigations on the turbulent circulation inside the cloud, and its effect on the lifetime of the suspended particles, are still rare and mostly attempted using off-the-shelf computational fluid dynamics (CFD) software.^13^ In this paper, we pursue a semi-analytical approach based on a mathematical description of the vortex dynamics. Key advantages of our semi-analytical model are: (i) it is validated against available experimental data on cough flow dynamics, (ii) it allows us to obtain a clear physical insight on the vortex dynamics, and how it affects the trajectories of the droplets, and (iii) it has a low computational expense, which makes it suitable for a variety of engineering applications. Indeed, according to Lai *et al.*’s analysis, semi-analytical particle cloud models are typically of the order of 1000 times faster than CFD, but still offer reasonably accurate predictions.^25^

Clinical evidence increasingly supports the case that airborne transmission via droplets carried by the expiratory cloud, whose key physical parameters are reported in Table I, plays a fundamental role in the high transmission rates of influenza, SARS-CoV-1, and SARS-CoV-2 (see Refs. 3, 20, and 21). Therefore, understanding the role of the complex vortex dynamics occurring in the expiratory cloud, and modeling the trajectories of the transported particles in a fast and efficient way, is very timely and important.

**TABLE I.** Typical parameters of violent respiratory events and relevant values found in the literature. ^a^Ambient air density at 23 °C and 19.1% relative humidity. ^b^Droplet density at 34 °C. Note that these values are indicative due to the large variability of results between different experimental studies.

Parameter	Symbol	Value	References
Cough velocity	…	(1.5–28.8) m s^−1^	14
Sneeze velocity	…	(10–100) m s^−1^	1, 2, 4, and 14
Exhaled volume	*V*_0_	(0.25 × 10^−3^–1.6 × 10^−3^) m^3^	6, 15, and 29
Volume fraction of droplets	*ϕ*	10^−7^–10^−5^	6 and 16
Ambient air density^a^	*ρ*_*a*_	1.172 kg m^−3^	6
Exhaled air density	$\rho_{c_{0}}$	0.98 kg m^−3^	6 and 17
Droplet density^b^	*ρ*_*d*_	993	6
Droplet diameter	*d*	(3–1000) *μ*m	6 and 18
Dynamic viscosity of air	*μ*	1.9 × 10^−5^ kg s^−1^ m^−1^	6
Cervical range of motion	*θ*_0_	(−58°)–(+54°)	19

**FIG. 1.**
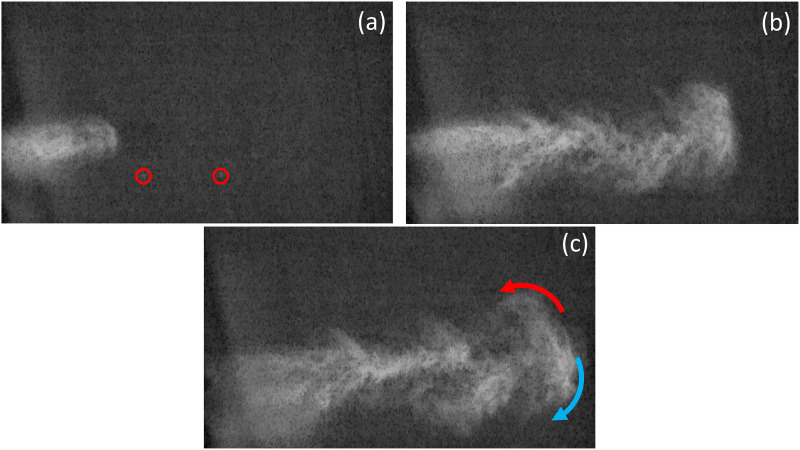
Snapshots of the propagation of a buoyant cloud vortex generated by a nozzle sprayer, analogous to a violent expiratory event.^10^ The initial flow speed at the nozzle is 2 m/s and the fluid temperature is 34 °C. The stills were recorded at 500 fps. (a) Time *t* = 200 ms. Soon after ejection, the larger droplets (some of which are visualized inside the red circles) are ahead of the cloud and follow a semi-ballistic trajectory. The moist cloud propagates in a self-similar fashion. (b) Time *t* = 850 ms. The jet is interrupted. The cloud expands by entraining ambient air, and a buoyant vortex structure emerges at the head of the cloud. (c) Time *t* = 1.1 s. The leading vortex is fully developed and is characterized by two branches. The upper branch (red arrow) has a positive vertical velocity and keeps the droplets in suspension. The lower branch (blue arrow) has a negative vertical velocity and allows the droplets to escape the cloud, eventually settling under the action of gravity. Note that the local trajectories inside the vortex strongly differ from the global trajectory of the cloud.

The recent work of Busco *et al.*^10^ shows that the flow dynamics of violent human respiratory events is analogous to that of fluid ejection from sprayers. Motivated by this analogy, we conducted experiments with a nozzle sprayer to visualize the vortex dynamics of the ejected cloud. Figure 1 shows several snapshots of the buoyant cloud ejected by a nozzle sprayer at different instants.

The nozzle ejects water droplets at 34 °C, in quiescent air at 24 °C ambient temperature and 64% relative humidity (RH). The speed of the ejected cloud at the nozzle, measured with a digital anemometer, is 2 m/s. At the back of the cloud, a backdrop is used to create a black-field effect and enhance visualization.^6^ The scene is lit by two LED lamps (each 1000 lm, 5600 K) and recorded via a Sony RX100 Advanced Camera at 500 fps. The images are captured in black-and-white to improve contrast and then post-processed using a morphological reconstruction algorithm on MATLAB, which enhances the bright regions surrounded by dark pixels.^22^ The analogy between this experiment and a real cough is strikingly evident by comparing the visualizations of Fig. 1 with the schlieren images of a cough taken by Tang *et al.*^23^ Figure 1 shows the two-stage dynamics of the process. At the onset of motion, the flow is a starting jet [Fig. 1(a)] that entrains ambient air as it travels, exhibiting a self-similar growth [Fig. 1(b)]. The larger droplets travel ahead of the jet following a semi-ballistic trajectory, whereas the medium-to-small size droplets remain inside the cloud. After the jet is interrupted, the hot and moist air visibly affects the dynamics of the system. An expanding and rotating spheroidal vortex structure develops at the front of the cloud, which is characterized by two counter-rotating branches [Fig. 1(c)]. At this stage, the dynamics is buoyancy dominated,^6^ and the circulation within the vortex determines the fate of the suspended droplets. The droplets captured by the downdraft flow in the lower branch escape the cloud and will eventually settle under the action of gravity. On the other hand, the droplets captured by the updraft flow in the upper branch remain airborne, until their downward settling speed exceeds the upward component of the velocity field inside the vortex.

As discussed in Ref. 29, the starting-jet phase is very short lived, lasting only a fraction of a second. On the contrary, the buoyant cloud dynamics evolves for hundreds of seconds. In this paper, we focus on the fate of the expiratory cloud after it is ejected. That is, we treat the expiratory cloud as a puff.^6^ We show that the dynamics of the expanding and rotating turbulent cloud can be modeled by adapting the theory of buoyant vortex rings with initial momentum. First, we use an integral approach to derive conservation laws for the expiratory cloud. Then, we embed the conservation laws into the mathematical model of an expanding Hill’s vortex^25^ to determine the velocity field. Furthermore, we reconstruct the trajectories of the exhaled droplets within the cloud using a Lagrangian particle-tracking model. We compare our results to the available experimental data, which show a very good agreement. The vortex present in the cloud is shown to have a significant effect on the droplet suspension, recirculating them within the cloud to varying degrees and extending their range further than previously predicted. We also study the role that initial conditions have on the range of the droplets, finding that decreasing the angle of projection can reduce the spread of the droplets by an order of meters. Finally, we discuss the importance of these findings in the context of informing public health policies and global information campaigns to slow down the spread of respiratory viruses.

## II. MATHEMATICAL MODEL

### A. Integral model

We start by characterizing the dynamics of the respiratory cloud by means of an integral model. The cloud is composed of the exhaled air, the mucosalivary droplets, and the entrained ambient air. Although the droplets are much denser than the cloud itself, the volume fraction contained within the cloud is on the order of 10^−7^ to 10^−5^ (see Table I). Therefore, it is safe to assume that the droplets play no significant role in determining the resulting trajectory of the cloud. As the cloud travels forward from the source, it entrains the ambient air causing it to grow in size and to follow a curvilinear trajectory (*S*, *θ*), as shown in Fig. 1. The growth is self-similar and proportional to the distance *S*(*t*) traveled by the leading vortex at time *t*, tan(*θ*) being the trajectory’s slope with respect to the horizontal (Refs. 6 and 24). Let us introduce a Cartesian reference system with orthonormal vectors ***i***, ***j***, and ***k*** oriented along the *x*-, *y*-, and *z*- axis, respectively, with ***k*** pointing upward. In the absence of ambient airflow, the mean trajectory of the cloud lies on the (*x*, *z*) plane (see Fig. 1). The momentum equation for the cloud reads^24^

$$\frac{dI}{dt}=\frac{d}{dt}\left[w_{c}V\left(\rho_{c}+C_{M}\rho_{a}\right)\right]=B-\frac{1}{2}\rho_{a}C_{D}Aw_{c}|w_{c}|.$$ (1)

In the latter, ***I***(*t*) = *I*_*x*_***i*** + *I*_*z*_***k*** is the cloud’s momentum, $w_{c}\left(t\right)=w_{c_{x}}i+w_{c_{z}}k$ is the velocity of the cloud’s centroid, *V*(*t*) the cloud’s volume, *ρ*_*c*_(*t*) the density, *C*_*M*_ the added mass coefficient, *ρ*_*a*_ is the ambient air density, ***B***(*t*) = (*ρ*_*a*_ − *ρ*_*c*_)*gV****k*** is the net buoyancy, where *g* is the acceleration due to gravity, *C*_*D*_ is the drag coefficient, and *A* is the projected cross-sectional area of the cloud perpendicular to ***w***_*c*_. In the case of a buoyant cloud in stagnant ambient fluid, *C*_*M*_ ≈ 0 and *C*_*D*_ ≈ 0 (Refs. 6, 24, and 25). Then, in scalar components on the vertical (*x*, *z*) plane, Eq. (1) simplifies to

$$\frac{dI_{x}}{dt}=0,\;\frac{dI_{z}}{dt}=B\left(t\right),$$ (2)

as in Ref. 6. Applying the initial conditions *I*_*x*_(0) = *I*_0_ cos(*θ*_0_) and *I*_*z*_(0) = *I*_0_ sin(*θ*_0_), where *I*_0_ = |***I***(0)| and *θ*_0_ = *θ*(0) are given, the system of Eq. (2) can be solved to give

$$I_{x}=I_{0}\cos\left(\theta_{0}\right),\;I_{z}=I_{0}\sin\left(\theta_{0}\right)+B_{0}t.$$ (3)

Note that the cloud buoyancy *B*(*t*) = *B*(0) = *B*_0_ remains constant, because the entrained air is neutrally buoyant^6^ and the volume of the droplets is negligible. Combining the terms in Eq. (3), the angle *θ*(*t*) is then given by

$$\theta =\tan^{-1}\left(\frac{B_{0}t}{I_{0}\cos\theta_{0}}+\tan\theta_{0}\right).$$ (4)

The cloud is observed to grow proportionally, at a rate *α*, to the distance *S* traveled from its source (see Fig. 1). Hence, the radius *r* of the cloud at time *t* is *r* = *αS*(*t*), as in Ref. 6. Moreover, the shape of the cloud observed in experimental studies with human participants is approximately ellipsoidal,^1,6^ with radius *r* and height *kr*, where *k* = 9/(4*π*). Therefore, the volume *V* of the cloud is

$$V=3\alpha^{3}S^{3}.$$ (5)

Since the initial ejected volume is non-zero, it is useful to define a virtual origin,^6^ as the point at which the radius would decrease to zero following Eq. (5). In all the simulations presented in this paper, the virtual origin is located at ***x*** = **0**.

As the cloud grows and its volume increases with the entrainment of ambient air, its density approaches *ρ*_*a*_. The density of the cloud *ρ*_*c*_ can be expressed as the sum of its initial volume *V*_0_ with density $\rho_{c_{0}}$, as a fraction of its current volume *V*, and the remaining volume fraction with density *ρ*_*a*_ (recall that the volume fraction of the droplets is negligible)

$$\rho_{c}=\rho_{c_{0}}\;\frac{V_{0}}{V\left(t\right)}+\rho_{a}\left(1-\frac{V_{0}}{V\left(t\right)}\right).$$ (6)

The speed of the cloud along its trajectory can be derived using its momentum, volume, and density as

$$w_{c}=\frac{dS}{dt}=\frac{I}{V\rho_{c}},$$ (7)

where $I=|I|=\sqrt{I_{x}^{2}+I_{y}^{2}}$.

Equations (2)–(7) give the properties of the cloud needed to compute its trajectory, internal velocities, and forces acting on the droplets (see Secs. II B and II C). Taking the time derivatives [excluding Eq. (4)] gives a system of coupled ordinary differential equations

$$\frac{dV}{dt}=9\alpha^{3}S^{2}\frac{dS}{dt},$$ (8)

$$\frac{d\rho_{c}}{dt}=\frac{1}{V}\left(9\alpha^{3}S^{2}\frac{dS}{dt}\left(\rho_{a}-\rho_{c}\right)\right),$$ (9)

$$\frac{dB}{dt}=g\rho_{a}\frac{dV}{dt}-g\frac{d\left(V\rho_{c}\right)}{dt},$$ (10)

$$\frac{dI_{z}}{dt}=B,$$ (11)

$$\frac{dS}{dt}=\frac{\sqrt{I_{z}^{2}+{\left(I_{0}\cos\theta_{0}\right)}^{2}}}{V\rho_{c}},$$ (12)

$$\begin{array}{ll} \frac{d\left(V\rho_{c}\right)}{dt}= & \;\left(3V_{0}\rho_{c_{0}}\alpha^{3}-3V_{0}\rho_{a}\alpha^{3}\right)\;\left(\frac{3S^{2}dS/dt}{V}-\frac{S^{3}dV/dt}{V^{2}}\right)\\ & +\;9\alpha^{3}\rho_{a}S^{2}\frac{dS}{dt}. \end{array}$$ (13)

The above system resembles that in Ref. 6, the difference being that here we neglect the minuscule volume fraction of the droplets entrained in the cloud. A fourth-order Runge–Kutta scheme was used to solve system (8)–(13) with a time step of Δ*t* = 0.001 s. This choice produces good convergence while maintaining a reasonable computational expense. We checked our numerical results against those of Ref. 6 and found excellent agreement. This confirms that the small volume fraction of the droplets does not affect the cloud dynamics.

### B. Velocity field

The characterization of the cloud provided by the integral model, including position, orientation, and size, is now used to compute the velocity fields of the internal vortex and the motion of the ambient fluid in the vicinity of the cloud. Based on the experimental evidence (see Fig. 1 and Refs. 1 and 6), we hypothesize that these velocity fields can be described mathematically with a buoyant vortex ring model. Turner^26^ was the first to use an expanding Hill’s vortex model to describe the flow induced thermally. In its original form, Hill’s vortex is an exact solution of the Euler equation^27^ describing a steady, non-expanding vortex ring. Turner’s extension of Hill’s original theory involved an *a priori* specification of the vortex radius as a function of time, to model air entrainment (see Refs. 24 and 26 for details). Following Turner, Lai *et al.*^24^ further expanded the model to consider a translating and expanding vortex, moving with a non-constant speed.

Here, we develop this theory further. Instead of assuming the angle *θ* to be constant, as Ref. 24 does, we embed conservation laws (8)–(13) and the resulting cloud trajectory (*S*, *θ*) into Hill’s original model. We assume that the motion of a fluid particle in the expanding and rotating buoyant vortex (see Fig. 1) is instantaneously the same as it would be near a fixed vortex of the same size. For the sake of simplicity, we introduce a reference frame local to the cloud, with its *x*′ direction aligned with the instantaneous velocity vector w_*c*_. A point ***x*** in the global reference system can be described in the local reference frame as ***x***′ via the transformation

$$\begin{array}{c} x^{\prime }=\left(x-x_{c}\right)\cos\theta +\left(z-z_{c}\right)\sin\theta ,\;y^{\prime }=y,\\ z^{\prime }=-\left(x-x_{c}\right)\sin\theta +\left(z-z_{c}\right)\cos\theta , \end{array}$$ (14)

where (*x*_*c*_, *z*_*c*_) is the instantaneous location of the cloud centroid on the vertical plane.

We now use the known position of the cloud in combination with its radius and angle of rotation, resulting from the integral model in Sec. II A, to calculate the components of the instantaneous velocity field at each point of the computational domain. For the points that lie within the boundary of the cloud, the instantaneous components of the flow field ***u***′(*x*′, *y*′, *z*′) = ($u_{x^{\prime }}^{\prime }$, $u_{y^{\prime }}^{\prime }$, $u_{z^{\prime }}^{\prime }$)(*x*′, *y*′, *z*′) at a particular time *t* are

$$u_{x^{\prime }}^{\prime }=\frac{-3w_{c}}{4}\left(\frac{4z^{\prime 2}}{r^{2}}+\frac{2x^{\prime 2}}{r^{2}}-\frac{10}{3}\right),$$ (15)

$$u_{y^{\prime }}^{\prime }=\frac{3w_{c}}{2r^{2}}\left(y^{\prime }x^{\prime }\right),$$ (16)

$$u_{z^{\prime }}^{\prime }=\frac{3w_{c}}{2r^{2}}\left(z^{\prime }x^{\prime }\right).$$ (17)

For the points that lie outside of the cloud boundary, the instantaneous components of the velocity field are

$$u_{x^{\prime }}^{\prime }=\frac{w_{c}r^{3}}{2}\frac{2x^{\prime^{2}}-z^{\prime 2}}{{\left(x^{\prime 2}+z^{\prime 2}\right)}^{5/2}},$$ (18)

$$u_{y^{\prime }}^{\prime }=\frac{3w_{c}r^{3}}{2}\frac{y^{\prime }x^{\prime }}{{\left(x^{\prime 2}+z^{\prime 2}\right)}^{5/2}},$$ (19)

$$u_{z^{\prime }}^{\prime }=\frac{3w_{c}r^{3}}{2}\frac{z^{\prime }x^{\prime }}{{\left(x^{\prime 2}+z^{\prime 2}\right)}^{5/2}}.$$ (20)

We remark that Eqs. (15)–(20) describe a flow field that is instantaneously the same as if the particles were in a fixed vortex (see Refs. 24 and 26). Therefore, Eqs. (15)–(20) can be described in the global reference system using the transformations

$$u_{x}=u_{x^{\prime }}^{\prime }\cos\theta -u_{z^{\prime }}^{\prime }\sin\theta ,\;u_{z}=u_{x^{\prime }}^{\prime }\sin\theta +u_{z^{\prime }}^{\prime }\cos\theta ,$$ (21)

whereas *u*_*y*_ = $u_{y^{\prime }}^{\prime }$ = 0 on the vertical plane *y* = 0.

### C. Droplet tracking

Once the velocity field is known, the droplet dynamics can be determined. The motion of each droplet is rendered by integrating the relevant particle tracking equation^24^

$$\rho_{d}V_{d}\frac{Du_{d}}{Dt}=F_{D}+F_{g}+F_{A}+F_{I},$$ (22)

where *D*/*Dt* denotes the Lagrangian derivative, *V*_*d*_ is the volume of the particle,

$$F_{D}=\frac{\rho_{a}\pi d^{2}C_{D}}{8}|u-u_{d}|\left(u-u_{d}\right)$$ (23)

is the drag force, *d* being the droplet diameter,

$$F_{g}=\left(\rho_{d}-\rho_{a}\right)V_{d}\;g$$ (24)

is the net gravitational force,

$$F_{A}=\rho_{a}V_{d}C_{M}\left(\frac{Du}{Dt}-\frac{Du_{d}}{Dt}\right)$$ (25)

is the added mass force, where $Du/Dt=\left(\partial /\partial t+u\cdot \nabla \right)u$ is the material derivative, and

$$F_{I}=\rho_{a}V_{d}\frac{Du}{Dt}$$ (26)

is the inertial force. Note that history forces can be neglected in a buoyant particle-laden flow.^25^ Substituting Eqs. (23)–(26) into Eq. (22) and performing some algebra, we obtain an expression for the droplet acceleration^25^

$$\begin{array}{ll} \left(1+C_{M}\frac{\rho_{a}}{\rho_{d}}\right)\frac{du_{d}}{dt}= & \;\frac{\mu \rho_{a}\pi dC_{D}\left(u-u_{d}\right)Re_{d}}{8V_{d}\rho_{d}}-\left(\frac{\rho_{a}}{\rho_{d}}-1\right)\;g\\ & +\;\frac{3}{2}\frac{\rho_{a}}{\rho_{d}}\frac{Du}{Dt}. \end{array}$$ (27)

This equation amends a sign mistake in the gravitational term in Refs. 24 and 25. In Eq. (27), the added mass coefficient *C*_*M*_ = 0.5 for a spherical droplet, whereas the drag coefficient *C*_*D*_ depends on the Reynolds number

$$Re_{d}=\frac{\rho_{a}|u-u_{d}|d}{\mu },$$ (28)

where *μ* is the cloud dynamic viscosity, according to

$$\begin{array}{c} C_{D}=\left(24/Re_{d}\right)\left(1+0.15Re_{d}^{0.687}\right)\;if\;Re_{d}<1000,\\ C_{D}=0.44\;if\;Re_{d}\geq 1000 \end{array}$$ (29)

(see Ref. 25). Still in Eq. (27), the material derivative is calculated analytically using the same approach as in Ref. 25. Equation (27) is solved at every time step to determine the droplet velocity and then to update its position.

## III. DISCUSSION

In this section, we first validate our model against the available experimental data and then discuss the key physical properties of the expiratory cloud. Since the diameter range of the exhaled droplets during human respiratory events is extremely wide (see Table I), here we focus on the 30 *μ*m–1000 *μ*m interval. Our choice to disregard smaller droplet sizes is motivated by the low average concentration of several respiratory viruses in human ejecta. For example, recent investigations have found that the average viral load of COVID-19 in sputum is 7 × 10^6^ copies/ml (see Ref. 28). This makes it unlikely to find viable virus in very small droplets (note that the volume of a 30 *μ*m droplet is only 1.4 × 10^−8^ ml).

### A. Experimental validation

**FIG. 2.**
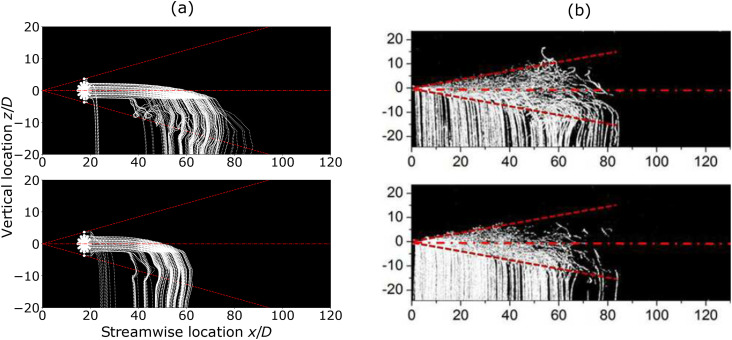
Streak plots comparing the mathematical model with the experimental results of Ref. 29, for *d* ∈ (57–68) *μ*m (top panels) and 96–114 *μ*m (bottom panels). The Reynolds number of the flow in both models is *Re* = 12 900. (a) Mathematical model. (b) Experimental results taken from Ref. 29 [J. Wei and Y. Li, “Human cough as a two-stage jet and its role in particle transport,” PLoS One **12**, e0169235 (2017). Copyright 2020 Author(s), licensed under a Creative Commons Attribution (CC BY) license].

Let us first compare our model results to the available experimental data. Wei and Li^29^ performed several experiments in a water tank to reproduce a cough flow. They also designed a careful scaling protocol, to make sure that the particle motion in the experiments is comparable to that of cough droplets in air. The particle (glass beads) payload was delivered via a small nozzle, equipped with a sediment-feeding system. Figure 2 shows a comparison between our model results and the streak pictures shown in Ref. 29 for 57 *μ*m–68 *μ*m diameter (top panels) and 96 *μ*m–114 *μ*m diameter (bottom panels), non-dimensionalized with respect to the nozzle diameter *D* = 2 cm (in physical scale). The initial conditions used in the mathematical model of Fig. 2(a) correspond to the developed cloud, once it has been driven away by the jet phase and starts evolving, carried by the flow.

Despite the feeding mechanism being much more complex (and the droplet number being much larger) in the experiments than in the model, the latter captures very well the key physical features of the system. The majority of the large particles (lower panels of Fig. 2) settle within 60*D* from the virtual source in both the mathematical and the physical model, though very few isolated particles reach about 80*D* in the latter. The smaller particles (upper panels of Fig. 2) have a larger penetration distance, as they are carried more easily by the cloud, and settle within 90*D* in both mathematical and physical models. The mathematical model clearly captures the droplets turning back on themselves, as they descend in the latter stages of their motion, drawing characteristic sickle-like trajectories [Fig. 2(a)]. These characteristic features, due to the circulation inside the vortex, also appear in the experimental streak pictures [Fig. 2(b)].

### B. Velocity field

**FIG. 3.**
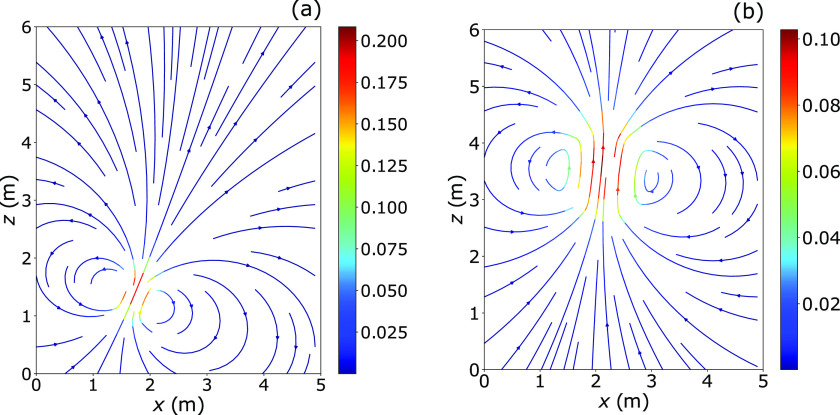
Snapshots showing the evolution of the velocity fields inside and outside of the expiratory cloud predicted by the vortex ring model. (a) *t* = 10 s. (b) *t* = 50 s. Parameters are: *I*_0_ = 1.32 × 10^−2^ kg m s^−1^, *B*_0_ = 2.3 × 10^−3^ N, *V*_0_ = 0.0012 m^3^, $\rho_{c_{0}}$ = 0.98 kg m^−3^, and *θ*_0_ = 23.8°.

Having validated our model, we now investigate the velocity field generated by violent expiratory events. Figure 3 shows stream plots for an expiratory cloud of parameters *I*_0_ = 1.32 × 10^−2^ kg m s^−1^, *B*_0_ = 2.3 × 10^−3^ N, *V*_0_ = 0.0012 m^3^, $\rho_{c_{0}}=0.98\;{kg\;m}^{-3}$, and *θ*_0_ = 23.9°, representing a typical cough. We chose these parameters to coincide with those of Bourouiba *et al.*,^6^ for the sake of comparison. The vortex shown in Fig. 3, given by the analytical formulae (15)–(20), features two counter-rotating branches, which resemble very closely those seen in the experiments depicted in Fig. 1. The upward curvature of the trajectory, an effect of buoyancy, also resembles the experimental dynamics. As time passes, buoyancy becomes dominant and the cloud tends to move further upward. At the same time, air entrainment causes the cloud’s speed to decrease. The overall dynamics of the cloud is therefore in agreement with the model results of Bourouiba *et al.*^6^

However, note that Bourouiba *et al.*’s model simplifies the settling dynamics of the droplets, as it assumes that each droplet group falls entirely out of the cloud once their settling speed *U*_*s*_ = *gd*^2^/(18*μ*)(*ρ*_*d*_ − *ρ*_*c*_) becomes greater than the cloud speed. On the contrary, our results show that the flow field inside the cloud is not uniform, and so the fate of each droplet is determined by the local velocity field in the cloud (including magnitude and direction). For example, in Fig. 3(b), a 40 *μ*m droplet (settling speed *U*_*s*_ = 4.55 × 10^−2^ m/s) would settle if located in the peripheral regions of the cloud. However, the same droplet would not settle if located close to the vortex core, where the upward vertical component of the velocity field exceeds the droplet’s settling speed. The way the expanding buoyant vortex affects the fate of droplet groups is analyzed in Sec. III C.

### C. Effects of vorticity on droplet range

In this section, we investigate the effect of the vortex structures present in the turbulent cloud (see Fig. 3) on the suspended droplets. We show that the leading vortex ring serves to re-suspend the droplets, as they begin to settle out of the cloud, effectively increasing their range.

**FIG. 4.**
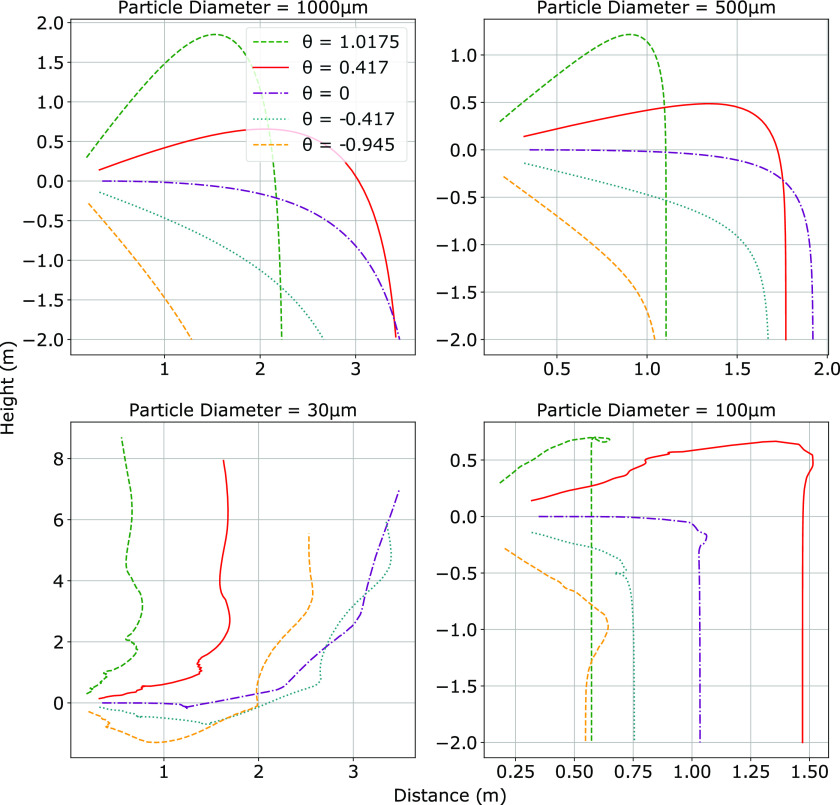
Trajectory of four discrete particle sizes for a cough projected at five different angles. Droplets with a diameter of 1000 *μ*m are shown in the top left plot, moving clockwise the remaining plots show 500 *μ*m, 100 *μ*m, and 30 *μ*m droplets. Ejection angles are in radians.

Figure 4 shows a series of plots comparing the trajectories of the droplets for various initial ejection angles *θ*(*t* = 0). The 1000 *μ*m and 500 *μ*m droplets follow the semi-ballistic trajectory observed in Ref. 6. Note that these droplets are easily projected past the common 2 m physical distancing guideline. Our results would instead suggest a 4 m physical distance, in agreement with the CFD model of Ref. 13. On the other hand, the 100 *μ*m and 30 *μ*m droplets are significantly more affected by the vortex present in the cloud, which causes them to reverse direction a number of times. The almost vertical downward trajectory of the 100 *μ*m droplets, as they settle out of the cloud and begin their descent to the ground, is a consequence of the stagnant environment outside of the cloud boundary. Lateral forces on the droplet are non-existent, allowing gravity and vertical drag to dominate the final descent of the droplets. The 30 *μ*m droplets appear to be influenced the most by the cloud trajectory, following it closely as they remain suspended for the entire duration of the simulation (200 s). These droplets are predicted to easily reach ceiling heights, in agreement with the earlier results of Ref. 6.

We now discuss the parametric dependence of the system on the initial ejection angle *θ*_0_. For diameters *d* > 30 *μ*m, the range is significantly decreased when *θ*_0_ is at the extremes of the typical cervical range of motion (CROM), i.e., 58° in extension and 54° in flexion.^19^ Coughs angled downward show the greatest decrease in range in all cases, except for the 30 *μ*m droplet. The horizontal ranges of 30 *μ*m droplets are minimized when projected upward; however, the decrease in the horizontal range is coupled with an increase in the vertical range. All of the 30 *μ*m droplets are capable of reaching ceiling heights, irrespective of the ejection angle. The model also shows that all of the droplets ejected at a neutral angle remain at an ejection height of between 1 m and 1.5 m, which indicates the worst-case scenario for an individual in close proximity to an infected person ejecting the droplets. A summary of these model predictions is shown in Table II.

**TABLE II.** Characteristics of droplet trajectories for different ejection angles.

Ejection	Particle	Max horizon.	Max vertical
angle (rad)	diameter (*μ*m)	distance (m)	height (m)
1.0175	1000	2.23	1.85
1.0175	500	1.11	1.22
1.0175	100	0.65	0.70
1.0175	30	0.77	8.70
0.417	1000	3.42	0.66
0.417	500	1.77	0.49
0.417	100	1.51	0.67
0.417	30	1.70	7.94
0	1000	3.46	0
0	500	1.92	0
0	100	1.06	0
0	30	3.49	7.02
−0.417	1000	2.67	−0.14
−0.417	500	1.67	−0.14
−0.417	100	0.76	−0.14
−0.417	30	3.40	5.93
−0.945	1000	1.28	−0.28
−0.945	500	1.04	−0.28
−0.945	100	0.64	−0.28
−0.945	30	2.57	5.54

**FIG. 5.**
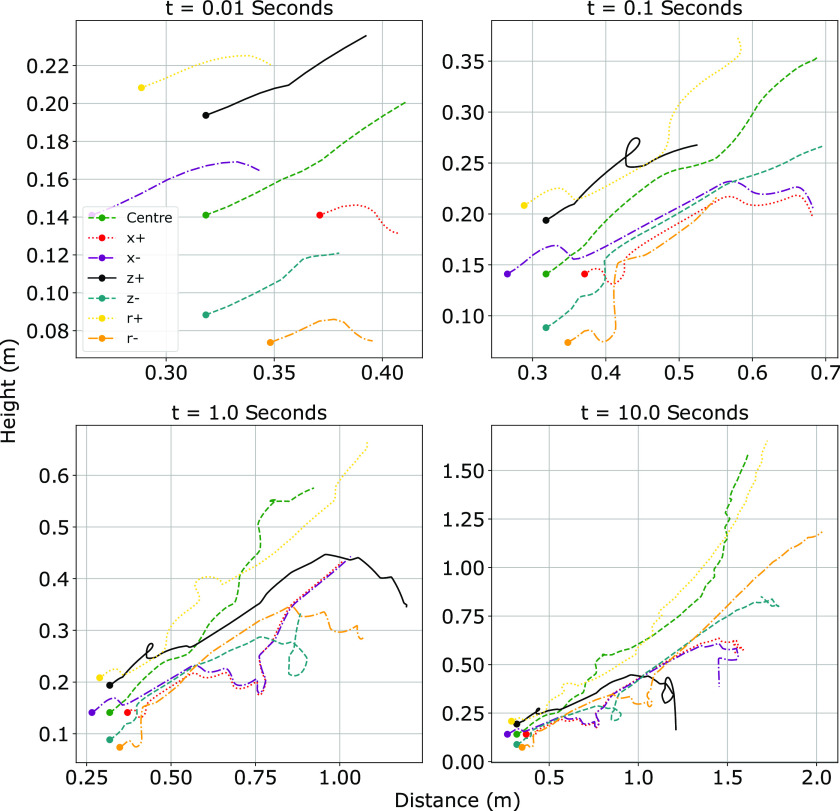
Snapshots of the trajectories of several 50 *μ*m droplets located at different initial positions in the expiratory cloud.

To further investigate the influence that the cloud has on the droplet trajectories, we simulate a cloud bearing multiple droplets of the same size. Figure 5 shows a series of plots depicting the time evolution of the paths taken by a group of 50 *μ*m droplets ejected at different positions in the cloud. At the 1 s mark, some of the trajectories have been forced to completely rotate through 360°, as the vortex circulates them within the cloud. Ten seconds after initiation, three of the droplets, originally located in the peripheral regions of the cloud, start to settle out of the cloud and begin their descent to the ground. The remaining droplets are separated into two distinct streams, either side of the centroid, by the vortex. Our results clearly show that the initial position of the droplets, as they are ejected in the respiratory cloud, plays a key role in determining their fate.

**FIG. 6.**
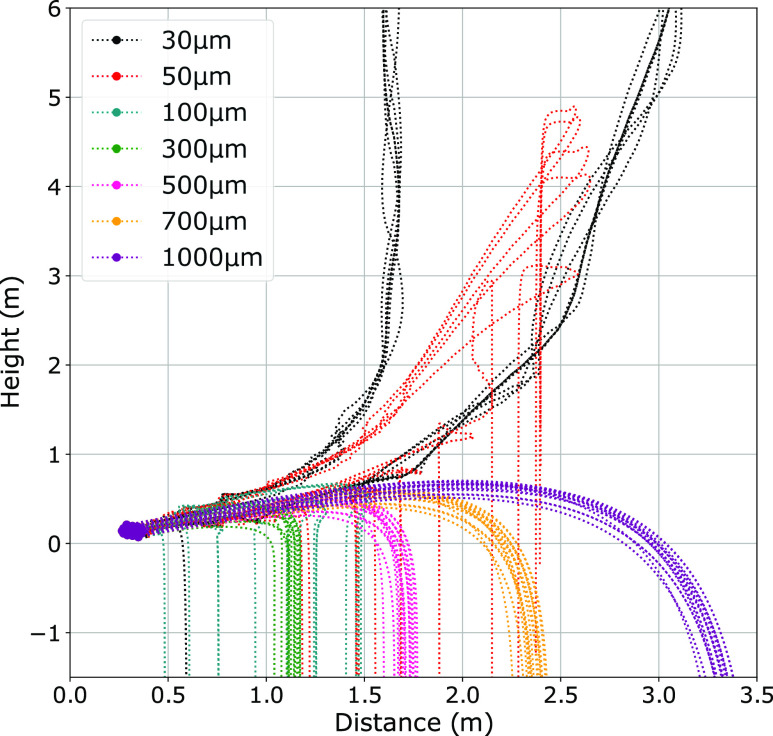
A poly-disperse cloud with droplets ranging from 30 *μ*m to 1000 *μ*m. Trajectories of the droplets over a time period of 200 s are plotted.

Finally, we investigate the behavior of a poly-disperse cloud containing droplets ranging from 30 *μ*m to 1000 *μ*m. Figure 6 shows the continuous fallout of droplets with diameter greater than 30 *μ*m, reinforcing our previous estimation using the local internal velocities (see Sec. II B). A few of the 50 *μ*m diameter droplets can be seen to recirculate within the cloud before settling out completely, indicating the vortex ability to extend the time the droplets remain airborne. Additionally, we see that the trajectories of same-size droplets get closer as the droplet diameter increases. For the 100 *μ*m droplets, a broad range in the horizontal distance is achieved. With droplets of diameter ≥300 *μ*m, the range becomes much closer and same-size droplets tend to cluster, as they become less and less affected by the circulation inside the cloud.

## IV. CONCLUSIONS AND RECOMMENDATIONS

In the majority of our analyses, the predictions made by our model suggest that the largest droplets consistently exceed the horizontal ranges of 2 m from the source before settling to the ground, in accordance with the recent findings.^1,3,6^ In some cases, the droplets are propelled in excess of 3.5 m (see Fig. 6). Therefore, guidelines suggesting 2 m physical distancing limits may not be adequate to prevent direct transmission via droplets of this size.

Moreover, in all of the cases explored, the smallest droplets modeled (*d* = 30 *μ*m) are also carried to great distances from the source. The paths taken by these small droplets are much more influenced by the flow field inside the expiratory cloud than the large droplets. The heights achieved by these droplets exceed 6 m above the source in the majority of cases. Our model shows that they take circa 40 s–60 s to reach a height of 4 m. At these heights, building ventilation systems will interfere with the dynamics of the cloud and could become contaminated.^1,6^ In all but one of the cases studied, the droplets with a diameter of 30 *μ*m remained suspended for the entire simulation period. Our findings contradict the fallout point predicted by Bourouiba *et al.*,^6^ which estimates that 30 *μ*m droplets completely fall out of the cloud at about *x* = 2.5 m from the source. Note that Bourouiba *et al.* did not consider the effects of vorticity. On the contrary, accounting for the vortex dynamics inside the clouds shows that the droplets are capable of traveling much further, before the turbulent vortex slows down sufficiently to allow them to begin rain out. For diseases capable of transmission via aerosol inhalation, these results begin to show the extent to which droplets may travel in relatively short timescales.

Another advancement of this model, upon the existing literature, is its ability to show the effect of the cloud vortex on the trajectories of same-size particles located at different points in the cloud. While traditional integral models would predict the same fate for all such particles,^6^ our results show that they split between the two sides of the cloud, as their trajectory is manipulated by each of the vortex branches, and therefore reducing their concentration in the air (see Fig. 3). Further analysis is required to explore the dilution of these tiny droplets, which undoubtedly have an effect on the likelihood of infection.

From our analysis showing how the droplet trajectory is affected by the ejection angle, it is apparent that directing the ejection downward significantly decreases the range for the majority of droplet sizes. The momentum imparted on the droplets at the time of release quickly drives them to settle on the ground. Behavioral and cultural changes in populations to direct coughs toward the ground, in addition to wearing face coverings, could help mitigate the risk of short-range direct transmission.

The position of the droplets relative to the centroid of the cloud at ejection plays an important role in determining their trajectories. This is increasingly apparent with smaller droplets, as they are more influenced by the internal turbulence. Droplets positioned at the bottom and rear of the cloud are more likely to settle out. This suggests that droplets ejected in the latter stages of exhalation are less likely to be far reaching. This finding highlights the need for further studies on the initial stages of violent respiratory events, to determine how fluid fragmentation contributes to the generation of droplets in different positions inside the cloud.^30^

We remark that this model is based on a number of simplifying assumptions, which allowed an engineering mathematics treatment of the problem, at a much lower expense than full CFD models. The boundary-layer modifications induced by physical boundaries (e.g., walls, ceilings, etc.), the influence of ambient air flow, and the dynamics of droplet evaporation inside the cloud, which are still poorly understood,^2^ need further investigation. These are the topics of our current research effort.

## DATA AVAILABILITY

The data that support the findings of this study are available from the corresponding author upon reasonable request.
